# Point-of-care ultrasound to complete physical exam and to reach the diagnosis in a young man with syncope

**DOI:** 10.1186/s13089-020-00176-5

**Published:** 2020-05-25

**Authors:** Fatemeh Rasooli, Mehrnoosh Aligholi Zahraie, Maryam Bahreini

**Affiliations:** 1 Department of Emergency Medicine, Tehran University of Medical Sciences, Tehran, Iran; 2 Prehospital and Hospital Emergency Research Center, Department of Emergency Medicine, Tehran University of Medical Sciences, Tehran, Iran

**Keywords:** Atrial myxoma, Syncope, Outflow obstruction

## Abstract

**Background:**

Cardiac syncope can result from an atrial myxoma due to outflow obstruction. Myxoma is the most common primary cardiac tumor that may cause sudden death and the nonspecific symptoms may make early diagnosis difficult.

**Case presentation:**

A 27-year-old man presented to our emergency department after two episodes of syncope and severe fatigue. He had no complaint of fever, weight loss, sweating, chest pain or dyspnea. Vital signs were within normal limits. A loud heart S1 was detected and normal neck veins. Other systemic examinations including neurological assessment were normal. Electrocardiography showed normal sinus rhythm. An obvious variability in heart rate was noticed on cardiac monitor changing by the patient’s position. Point-of-care ultrasonography (PoCUS) showed a large hyperechoic lesion with a well-defined stalk originating from the left atrium (LA). Thus, the patient was transferred to a cardiac surgery center for surgical intervention. Histopathology reported an LA mass compatible with myxoma.

**Conclusions:**

Emergency physicians should be familiar with the vague presentations of cardiac tumors to improve patient outcomes. It is beneficial to take advantage of bedside ultrasound for prompt diagnosis and subsequent treatment.

## Background

Syncope is a sudden temporary loss of postural tone and consciousness resulting from transient decreased cerebral perfusion, seen in nearly 35% of the general population. It is responsible for high rates of emergency ward visits and hospital admissions annually [1]. The most important causes of syncope include cardiac, neurologic and metabolic disorders, and medication side effects. Moreover, cardiac syncope may result from obstructive, ischemic or conductive heart diseases. This is typically secondary to either a structural or mechanical cardiac problem or due to arrhythmias that alter electrical conduction throughout the myocardium and the latter are recognized as the main cardiac mechanisms for syncope [1]. Atrial myxoma or other cardiac tumors should be considered for prompt diagnosis and treatment regarding the nonspecific symptoms such as weakness, syncope or lightheadedness. In this case, syncope occurred due to an atrial myxoma. Bedside ultrasonography was very helpful to achieve the diagnosis and fast decision-making in this patient.

## Case presentation

A 27-year-old man presented to our emergency department with a history of two episodes of syncope and severe fatigue. He had no complaint of fever, weight loss, sweating, chest pain or dyspnea. Past medical and habitual history was negative. Vital signs were blood pressure 129/75 mmHg, heart rate 75 beats/min, respiratory rate 18 breath/min and oxygen saturation 99% on room air. He was slightly confused with a fluctuating pattern. A loud heart S1 and normal neck veins were observed. Other systemic examinations including neurological assessment were normal.

**Fig. 1:**
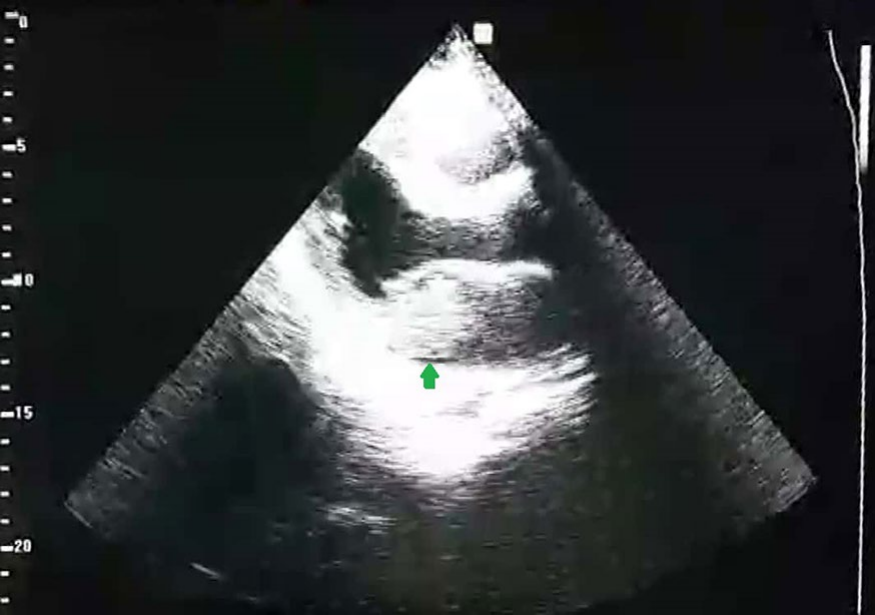
Transthoracic echocardiography showed a large hyperechoic lesion, located in left atrium in the systolic phase (arrow)

**Fig. 2:**
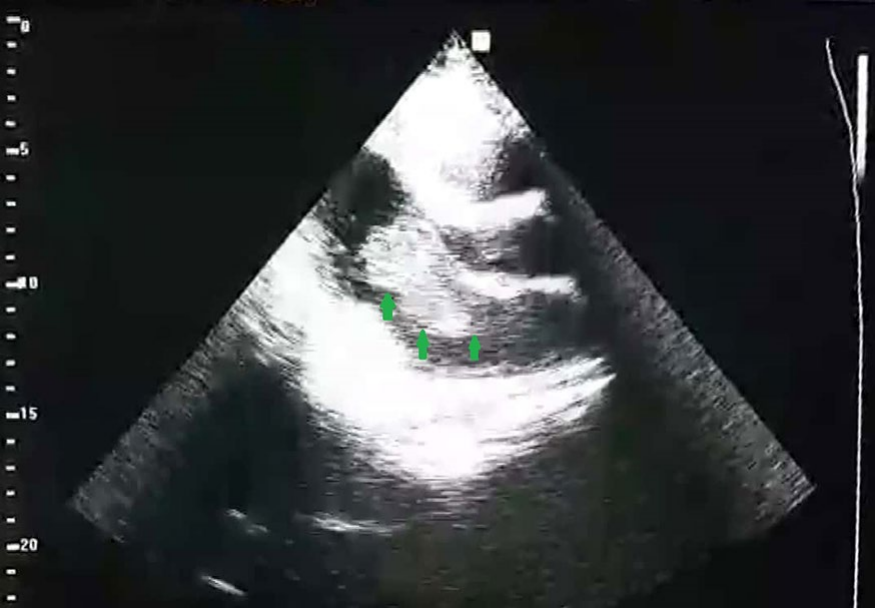
The highly mobile mass floating into the left ventricle during the diastolic phase (showed by arrows)

Laboratory findings were in normal range. Electrocardiography showed normal sinus rhythm. Brain computed tomography scan and chest X-ray did not show any abnormal findings. Interestingly, an obvious variability in heart rate was noticed on cardiac monitor changing by the patient position. Point-of-care ultrasonography (PoCUS) showed a large hyperechoic lesion with a well-defined stalk originating from left atrium (Figs. 1, 2). Thus, the patient was emergently transferred to a cardiac surgery center for surgical intervention during which a 60-mg mass was removed from his left atrium compatible with myxoma.

## Discussion

Atrial myxoma is the most common primary cardiac tumor that may lead to sudden death [2–4]. Because of the nonspecific symptoms such as weakness and syncope, early diagnosis may be difficult and the symptoms vary according to the tumor location in atrium or ventricle [2, 3]. In sporadic cases, atrium is the main involved heart chamber [4]. Myxoma is usually located in left atrium [5].

Dyspnea is usually the most common reported symptom [3, 4]. Furthermore, the presentation may be an ordinary symptom such as chronic palpitation lasting for 3 months reported by Prousi et al. [6]. Torregrossa et al. reported a case of a 61-year-old man with 1-year history of chronic cough and bilateral low-extremity swelling suspected initially by emergency ultrasound as an atrial mass despite a chronic course [7]. Comparatively, our patient was presented with acute-onset symptoms. Another finding in this case was the younger age in comparison with other studies that reported the mean age of nearly 50-years for myxoma [5, 8]. Another study reported unusual manifestations such as acute bilateral limb ischemia in an 18-year-old female by Mathew et al. [9].

However, some more complex situations are also reported such as a simultaneous existence of right coronary artery and left atrial myxoma [10]. Also interestingly, it may be seen as an incidental finding during chest computed tomography in a case with motor vehicle collision reported by Ali et al. [11].

Echocardiography is the method of choice for the diagnosis of atrial myxoma [12]. Despite various clinical presentations, bedside ultrasound and echocardiographic assessment may be beneficial in suspected cases in the emergency department as shown by Raja Rao et al. [13]. In this context, a mobile mass with a narrow stalk in a heart chamber may be suggestive for myxoma or possibly a thrombus [14]. In spite of various clinical manifestations and the need for different therapeutic surgical modalities, the final long-term prognosis is usually fair without significant unfavorable outcomes [15, 16] although there are some reports about fatal outcomes and recurrence after operative surgery [4, 8].

## Conclusion

Overall, it should be emphasized that emergency physicians should be familiar with the vague presentations of cardiac tumors to improve final outcomes in these patients [9]. It is crucial to take advantage of bedside ultrasound for prompt diagnosis and subsequent treatment (Additional file 1).

## Supplementary information

**Additional file 1.** Parasternal long-axis echocardiogram movie showing a highly-mobile mass floating into the left ventricle during the diastolic phase.

## Data Availability

None.

## Abbreviations

PoCUS: Point-of-care ultrasonography
LA: Left atrium
